# [Retracted] AdipoR1‑mediated miR‑3908 inhibits glioblastoma tumorigenicity through downregulation of STAT2 associated with the AMPK/SIRT1 pathway

**DOI:** 10.3892/or.2024.8697

**Published:** 2024-01-04

**Authors:** Xiangming Liu, Jinglong Chen, Jinqian Zhang

Oncol Rep 37: 3387–3396, 2017; DOI: 10.3892/or.2017.5589

Following the publication of this paper, it was drawn to the Editor's attention by a concerned reader that certain of the western blotting data shown in Figs. 3B and 9, and the migration assay data shown in Fig. 6C, were strikingly similar to data that had already appeared in other publications written by different authors at different research institutes. Owing to the fact that the contentious data in the above article had already been published prior to its submission to *Oncology Reports*, the Editor has decided that this paper should be retracted from the Journal. The authors were asked for an explanation to account for these concerns, but the Editorial Office did not receive a reply. The Editor apologizes to the readership for any inconvenience caused.

